# Mapping Road Surface Type of Kenya Using OpenStreetMap and High-resolution Google Satellite Imagery

**DOI:** 10.1038/s41597-024-03158-7

**Published:** 2024-04-03

**Authors:** Qi Zhou, Zixian Liu, Zesheng Huang

**Affiliations:** 1 School of Geography and Information Engineering, China University of Geosciences, Wuhan, 430074 China; 2 International Research Center of Big Data for Sustainable Development Goals, Beijing, P.R. China

**Keywords:** Geography, Sustainability

## Abstract

Identifying road surface types (paved or unpaved) can ensure road vehicle safety, reduce energy consumption, and promote economic development. Existing studies identified road surface types by using sensors mounted on mobile devices and high-resolution satellite images that are not openly accessible, which makes it difficult to apply them to large-scale (e.g., national or regional) study areas. Addressing this issue, this study developed a dataset of road surface types (paved and unpaved) for the national road network of Kenya, containing 1,267,818 road segments classified as paved or unpaved. To accomplish this, this study proposes a method that integrates crowdsourced geographic data (OpenStreetMap) and Google satellite imagery to identify road surface types. The accuracy, recall, and F1 score of the method were all above 0.94, validating the effectiveness of the method. The data sources of the method are freely available, and the method may be applied to other countries and regions. The dataset developed based on the method can provide data support and decision support for local governments to improve road infrastructure.

## Background & Summary

Road surface type (e.g., paved or unpaved) not only affects vehicle safety and energy consumption, but also affects road accessibility and socio-economic development^1–3^. In many regions of the world, there is great potential for economic development, but they are constrained by poor road surface quality, resulting in limited accessibility^4,5^. To address this challenge, the World Bank has adopted the Rural Access Index (RAI), which is the proportion of people living within 2 km of an all-season road^6^. RAI has also been included in the United Nations Sustainable Development Indicator (9.1.1) in 2017. An ‘all-season road’ was defined by the World Bank as a road that can be used by rural transport vehicles throughout the year^7^. Generally speaking, paved roads can be used all year round, while unpaved roads may be affected by rain and snow and cannot be used all year round. According to an existing study^8^, the length of unpaved roads in Africa is approximately 1.68 million kilometers, accounting for a 78% of total road length of this region. Using Kenya as an example, road transport constitutes the predominant mode of transportation in this country, representing over 90% of the national passenger and freight volumes^9^. Nevertheless, more than 50% of the country’s roads in Kenya remain unpaved^9^, leading to transportation inconveniences, decreased efficiency, and hindrances to attracting investments, thereby constraining local socio-economic development^10^. Therefore, there is an urgent need for the local government to deploy rapid and effective means to monitor the road surface types, because identifying paved and unpaved roads not only helps to assess the RAI, but also can provide decision support for local governments to improve road infrastructure.

Currently, there are many studies on identifying road surface types. For example, Abbondati *et al*. developed a system that uses a GPS receiver and a triaxial accelerometer mounted on a mobile device to detect road surface types^11^. De Blasiis *et al*. proposed an algorithm to determine road surface roughness and road defect types, namely potholes and swells/shoves, based on 3D point cloud data collected by a vehicle-mounted radar system^12^. Shon *et al*. developed a self-monitoring road management system to detect road surface types (paved and unpaved) on the Korea-Japan Highway^13^. Staniek developed a system to collect data from smartphone users and used the city of Tychy in Poland as an example to assess road defects, namely unpaved roads categorized into gravel and earth roads, by analyzing the dynamics of vehicle motion^14^. These studies are all based on sensors mounted on mobile devices to monitor road surface types.

Furthermore, scholars have proposed methods for assessing road surface types based on satellite remote sensing imagery. Kavzoglu *et al*. utilized IKONOS satellite remote sensing data with a spatial resolution of 4 meters to identify the quality of road surfaces in the Istanbul region of Turkey (categorized as good, medium, and bad)^15^. Karimzadeh *et al*. employed high-resolution satellite imagery from a multispectral VHR Pléiades-1B with a spatial resolution of 0.5 meters to identify road surface types (paved and unpaved) in the southeastern areas of South Africa’s Gauteng and Northwest provinces^16^. Brewer *et al*. constructed a dataset for recognizing road surface quality in Virginia, comprising high, medium, and low-quality categories, using high-resolution (0.3 meters) satellite images supplied by the VBMP (Virginia Geographic Information Network’s Virginia Base Map Project), convolutional neural networks and transfer learning strategies^17^. Workman *et al*. utilized non-public high-resolution satellite imagery and a ground truth dataset manually annotated by local road management authorities in Tanzania to establish a classification dataset encompassing four categories of road surface quality: good, fair, poor, and very poor. This dataset was then applied to identify road surface quality in the Kilosa region of Tanzania^18^.

However, there are still several shortcomings in the existing methods. First, the method of integrating sensors into mobile devices to monitor road surface type requires field data collection. However, this process is not only time-consuming but also difficult to implement in a large-scale area (such as national scale)^19^. Although satellite remote sensing imagery are widely regarded as a powerful tool for conducting large-scale studies, identifying road surface type can be challenging with medium and low-resolution remote sensing imagery. Thus, most of existing studies used high-resolution remote sensing data (e.g., below 1 meter), but these data are not open to the public, it is therefore hard to utilize the data into a different study area. Furthermore, object extraction and classification from remote sensing images often demands a substantial number of training samples. Notably, training samples derived from a singular region or dataset may not be universally applicable to other regions. Thus, the ongoing research challenge is to develop a method for automatically and adaptively identifying road surface type at a large scale.

In recent years, geographic data, e.g. OpenStreetMap (OSM), edited and updated by global volunteers has been considered as an important source of obtaining global geographic information. With the advantages of free access, global coverage, and rich geographic features, OSM data provides the possibility of obtaining training samples for road surface type at the national and even global scale^20,21^. On the other hand, high-resolution Google satellite imagery not only provides rich information on the earth but also has the feature of global open access.

Hence, this study proposes a method for identifying road surface types through the integration of OpenStreetMap road data and high-resolution Google satellite imagery. Specifically, this research utilizes road surface type labels marked by volunteers in the OSM road data as prior knowledge and combines them with corresponding Google satellite images for the automatic generation of machine learning training samples. Subsequently, various convolutional neural networks are compared for their accuracy in identifying road surface types. The optimal network is selected for training, and the best model is applied to the images corresponding to sampling points on every road in Kenya, resulting in the creation of the road surface type dataset for the country. Our primary contributions include:

1. A method to identify road surface type by integrating OSM road data and high-resolution Google satellite imagery is proposed.
2. Based on the method, a dataset for the road surface type of Kenyan roads has been developed, encompassing the surface types (paved and unpaved) of 1,267,818 road segments in this country. This dataset can serve as decision support for the local government in enhancing road infrastructure.

## Methods

### Overview

**Fig. 1:**
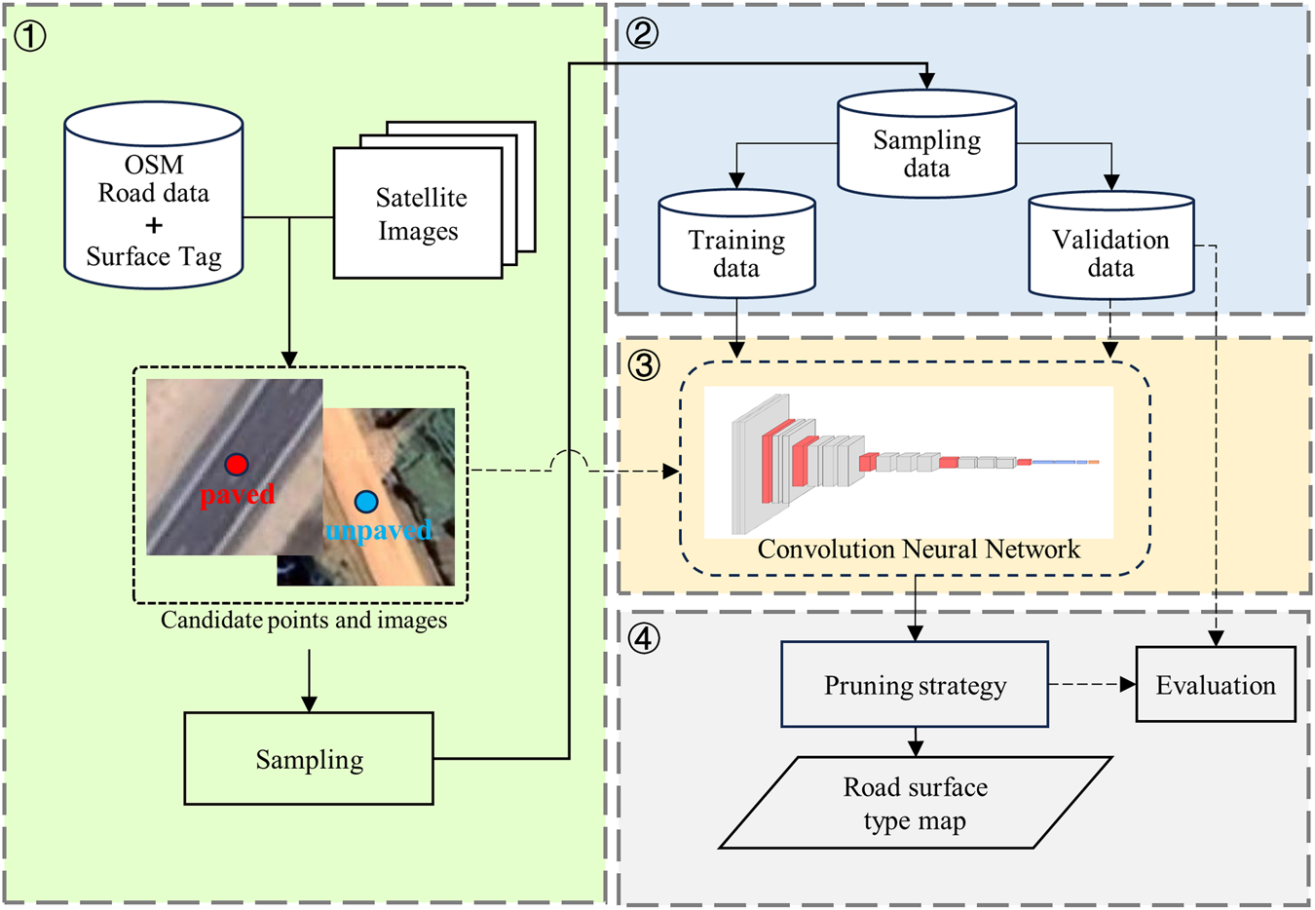
The workflow of our approach.

Firstly, candidate points across the country-Kenya were generated based on the OSM road data, and the corresponding Google satellite images for each point were also used. Subsequently, a specific number of sampling points were randomly selected from these candidate points, with each sampling point corresponding to a road segment containing OSM road surface type (paved or unpaved). Following this, the convolution neural network was employed to train 70% of the sampling points, while the remaining 30% were reserved for validating the model’s performance. Finally, the trained model was applied to all candidate points in Kenya, using a pruning strategy to determine road surface type for each road. Figure 1 illustrates the workflow for creating the road surface type dataset for the Kenyan road network.

### Data collection

Initially, OSM road data (© OpenStreetMap https://www.openstreetmap.org/copyright) till January 2023 were downloaded from the Google Cloud Platform using Python scripts. The data was used because an existing study has found that in Kenya, the OSM road data has the highest completeness and positional accuracy than other road datasets^22^. Then, the acquired OSM road data underwent topological checks by using the ‘topology check’ in ArcGIS, in order to make sure that each pair of intersected road segments has an intersection point. Additionally, the ‘surface’ tags in the OSM road data were reclassified into two categories: paved roads and unpaved roads (Table 1) with reference to:https://wiki.openstreetmap.org/wiki/Key:surface.

**Table 1:** Reclassifying OSM surface tags into paved and unpaved roads.

OSM Surface Tag	Reclassification
Paved, asphalt, concrete, concrete: plates, paving stones, pebblestone	Paved
Unpaved, dirt, ground, earth, sand, wood	Unpaved

Then, candidate points were generated along the OSM road segments at intervals of 100 meters. If a road segment’s length was less than 100 meters, the midpoint of the segment was designated as the candidate point. Given that a road intersection often connects multiple road segments, each with potentially different road surface types, candidate points located within 15 meters of a road intersection were excluded. This decision was based on the spatial resolution of a single Google satellite image being 0.1 meters^23^, with pixels measuring 200 × 200, resulting in a maximum distance from the image center point to the edge of approximately 0.1 × 100×$\sqrt{2}$, which approaches 15 meters. Subsequently, the Google satellite images (Maps Data ©2023 https://www.google.com/maps/) corresponding to each candidate point were processed online using Python scripts within the QGIS software, amassing a total of 3,304,609 candidate points, each associated with a corresponding Google satellite image.

### Training and validation data preparation

5,000 sampling points marked as ‘paved’ and 5,000 sampling points marked as ‘unpaved’ were randomly extracted from the candidate points that contain the OSM road surface type tag, which was achieved using the ‘random.sample’ function in python. Each sampling point was visually interpreted by three different operators as paved or unpaved, using Google satellite map and Google street view map. The reference category of each sampling point was finally determined using a voting method. Then, 70% (7,000) of these 10,000 sampling points were used for training and 30% (3,000) for validation, and the ratio between ‘paved’ and ‘unpaved’ sampling points was set as 1:1 in both the training and validation datasets.

### Model training and selection

Existing studies have shown that convolutional neural networks are superior to traditional machine learning methods such as support vector machine and random forest in high-resolution remote sensing imagery classification^24–26^. However, there is a wide variety of convolutional neural networks (CNNs). Thus, six different CNNs were explored, including VGG-16^27^, VGG-19^27^, Inception-v2^28^, Inception-v3^28^, ResNet-34^29^, and ResNet-50^29^. These models were chosen because they have been widely used and compared for determining an optimal model^30–32^. After experimentation, the VGG-16 model was ultimately chosen for its superior classification performance.

However, training a VGG-16 model usually requires a large amount of training data and takes a lot of time^33^. To speed up the training, this study draws on the idea of transfer learning proposed by existing studies: that is, introducing pre-trained model parameters to help model learning faster^34^, Specifically: the VGG-16 network has been pre-trained on a large computer classification dataset ImageNet^35^, which contains 1,000 categories and more than 1,000,000 images. However, ImageNet does not include the two categories of ‘Paved’ and ‘Unpaved’. Therefore, this study used the convolutional neural network trained on ImageNet, and set the number of outputs of the softmax layer to 2, and then trained it based on our 7,000 samples. Certain parameters must be configured before initiating the training of the network, such as the learning rate, weight decay, batch size, and the optimizer type^27^. For this study, the learning rate (to control the rate at which an algorithm updates or learns the values of a parameter estimate), the weight decay (to prevent model overfitting) and the batch size (the number of samples utilized in one iteration) were set at 0.0001, 0.001, and 64 respectively. These parameters were determined after trying different combinations, in order to achieve the best performance efficiently. The Adam optimizer was selected^27^. Furthermore, the model was implemented using the Pytorch framework and underwent training on an NVIDIA RTX 4070 Ti graphics card with 12GB of memory.

### Application and pruning

**Fig. 2:**
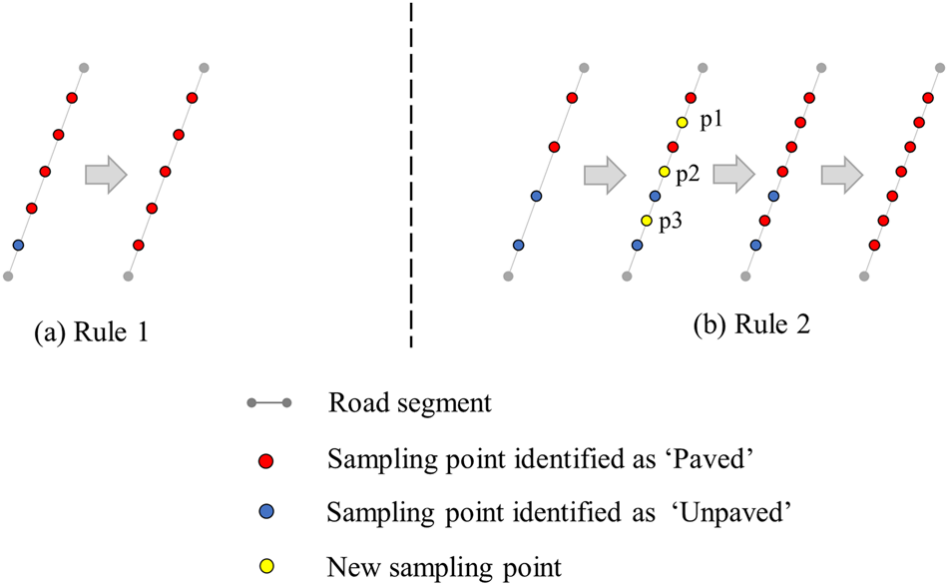
A schematic map of the pruning strategy.

The trained network was applied to the Google satellite images corresponding to all the candidate points in Kenya, and used to determine the road surface type of each candidate point. Considering that there might be errors in the classification results of the candidate points, a pruning strategy was introduced. We assumed that the road surface type of the same road segment was the same, and then determined the road surface type of each road segment according to the following two rules (refer to Fig. 2):

Rule 1: The road surface type of the majority of the candidate points on a road segment is taken as the road surface type of that road segment (Fig. 2a);

Rule 2: While the number of candidate points classified as paved and unpaved on a road segment is the same, new points (‘p1’, ‘p2’, and ‘p3’ in Fig. 2b) is added at the midpoint of the adjacent candidate points (i.e., resulting in an odd number of candidate points), and then Rule 1 is used (Fig. 2b).

### Results evaluation

The effectiveness of this method was evaluated from two aspects. First, the effectiveness of the model was evaluated using widely used metrics, namely precision, recall and F1 score, for the randomly extracted 10,000 sampling points (see Training and Validation data preparation section). Specifically, the following three scenarios were considered:

Scenario I: The quality of OSM surface tags was evaluated based on all 10,000 sampling points.

Scenario II: The effectiveness of the trained model (without the pruning strategy) was evaluated based on 3,000 validation set sampling points.

Scenario III: The effectiveness of the trained model (with the pruning strategy) was also evaluated based on 3,000 validation set sampling points.

Furthermore, existing studies have reported that there is a strong correlation between the length of paved roads and economic development indicators (such as Gross Domestic Product, GDP)^36,37^. Therefore, the administrative division data of Kenya at the county level (https://gadm.org/) and the statistical data of GCP (Gross County Product) for each county (https://www.knbs.or.ke/) were obtained. Then, the administrative division data of Kenya at the county level and the road surface type dataset developed in this study were overlaid, and the length of paved roads for each county was calculated. Finally, the correlation between road paving length and GCP was calculated using the Pearson correlation coefficient.

## Data Records

The road surface type dataset of Kenya consists of: Line vector data in Esri Shapefile format, totalling 1,267,818 road segments and has been made public under Figshare^38^. Each road segment contains a unique ID number and a road surface type classification (Table 2). The data is projected using the World Geodetic System (WGS) 84 and the Pseudo-Mercator projection coordinate system (EPSG: 3857).

**Table 2:** The fields in road surface type dataset of Kenya.

Field	Description	Type
ID	The id of each road segment	Integer
surface	The classification of road surface type (i.e., ‘Paved’ or ‘Unpaved’)	String
start_x	The value of the x coordinate of the starting point of each road segment	double
start_y	The value of the y coordinate of the starting point of each road segment	double
end_x	The value of the x coordinate of the ending point of each road segment	double
end_y	The value of the y coordinate of the ending point of each road segment	double

## Technical Validation

### Evaluation results

As shown in Table 3, for scenario I, all the metrics of the OSM road surface tags exceed 0.95. This indicates that most of the OSM road surface tags are accurately classified by the volunteers, and on the other hand, it also indicates that the OSM road surface tags are reliable for the machine learning model. Moreover, the performance metrics of the VGG-16 trained model all exceeded 0.90, validating the effectiveness of the proposed method. For scenario II, metrics for the ‘Unpaved’ category were a little higher, possibly due to a relatively larger number of training samples for unpaved roads compared to paved roads, making it easier for the model to learn features specific to unpaved roads. The metrics after the correction with the pruning strategy showed improvements compared to Scenario II, especially for the ‘Paved’ category, confirming the effectiveness of the pruning strategy (see scenario III).

**Table 3:** Evaluation results of the three scenarios.

Scenario	Class	Precision	Recall	F1 score	Support*
I	Paved	0.95	0.98	0.97	4,817
	Unpaved	0.96	0.98	0.97	5,183
II	Paved	0.91	0.90	0.91	1,427
	Unpaved	0.94	0.92	0.93	1,573
III	Paved	0.93	0.93	0.93	1,427
	Unpaved	0.92	0.95	0.93	1,573

Support* represents the number of sampling points.

### Evaluation by correlation analysis

**Fig. 3:**
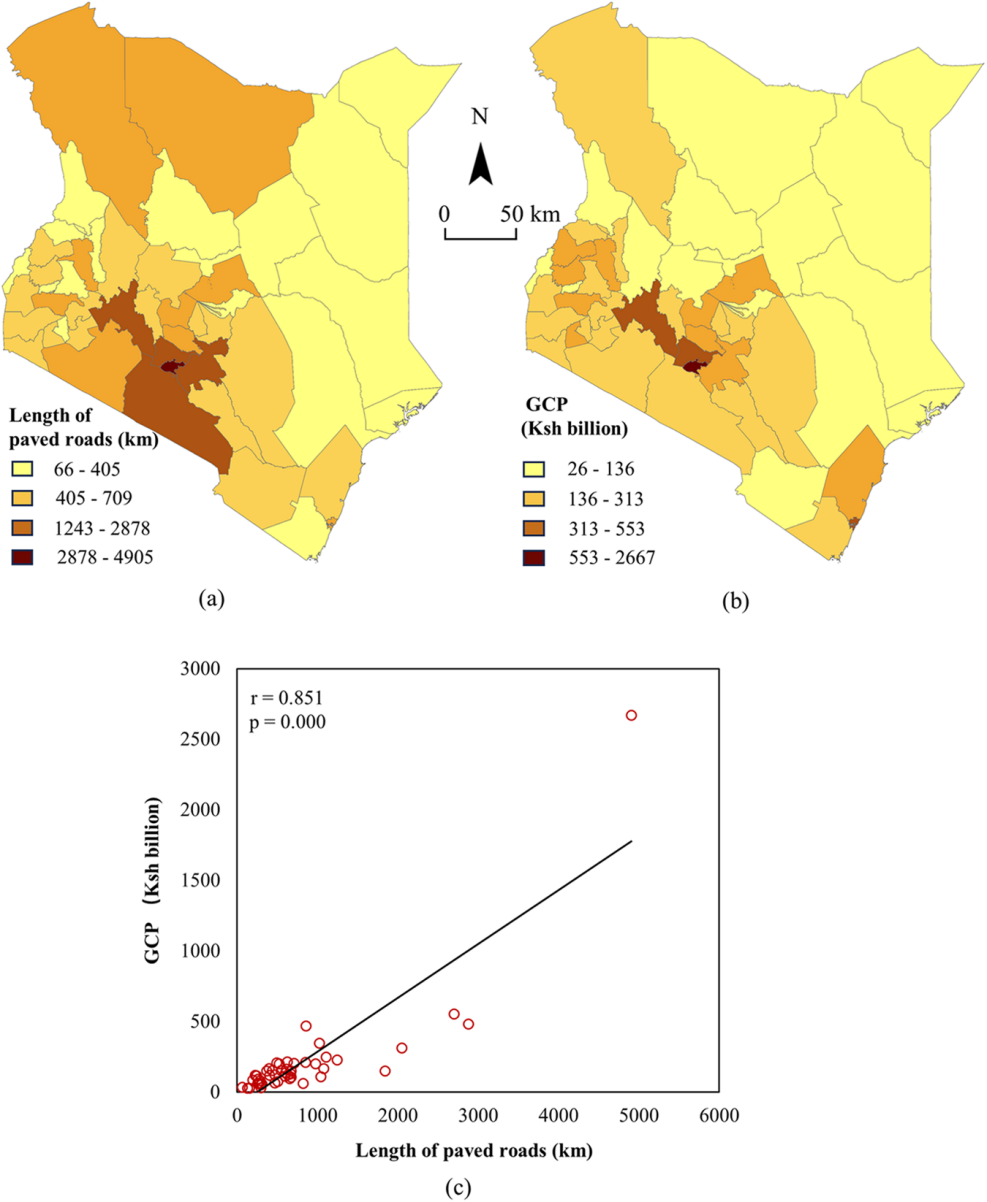
Results of the road surface type map of each county of Kenya. (**a**) shows the calculated length of paved roads for each county in Kenya, (**b**) shows the GCP data for each county in Kenya, and (**c**) shows the scatter plot of the correlation between road paving length and GCP for each county in Kenya.

The correlation analysis results between road paved length and Gross County Product (GCP) are illustrated in Fig. 3.

As shown in Fig. 3, there are more paved roads in the southwest region of Kenya, and the GCP in that region is also relatively high, showing consistency. The Pearson correlation coefficient *r* between the length of paved roads and GCP for each county in Kenya exceed 0.84, and is significantly correlated (p < 0.05), indicating a correlation between economic development in the region and road surface types. This finding is consistent with the existing research results^36,37^, validating the effectiveness of the method proposed in this study.

### Limitations and future directions

The limitations of this study are as follows:

1. This study classified road surfaces into paved and unpaved, however, road surfaces can also be subdivided into ‘asphalt’, ‘concrete’, ‘sand’ and ‘dirt’ etc. This study did not consider finer classifications because: on the one hand, it is difficult to distinguish the finer categories of road surfaces from Google satellite images^39^; on the other hand, the paved roads in Kenya are mainly asphalt, while the unpaved roads are mainly dirt, and the OSM road lengths of other surface materials are very low (less than 3%). Nevertheless, future studies can consider using field surveys to obtain the finer categories of road surfaces, and verify whether the proposed method can automatically identify these categories.
2. This study used the VGG-16 model to train the sampling points, and all the indicators exceeded 0.90, validating the effectiveness of our method. Despite this, this study used a basic model, and future studies can consider adding modules such as spatial attention mechanism, channel attention mechanism, etc., to verify whether they can improve the effectiveness of the method. On the other hand, Google satellite images are obtained by different sensors at different times^40^, which may cause large differences in hue and texture of the satellite images of the same area^23^. Some Google satellite images of roads may also be blocked by trees or buildings^39^. Although the pruning strategy helped to improve the identification results of road surfaces in this study, future studies still need to consider using other high-resolution remote sensing images or street view data as auxiliary, to improve the effectiveness of the method.
3. The method of this study was only applied to Kenya because Google street views, used to determine the reference classification of each sampling point, were not available for most countries in Africa. Despite of this limitation, OSM data and Google satellite imagery can be used for free worldwide, and are being continuously updated. In the future, we will apply this method to other countries and regions in the world to verify the effectiveness of the proposed method.

## Usage Notes

**Fig. 4:**
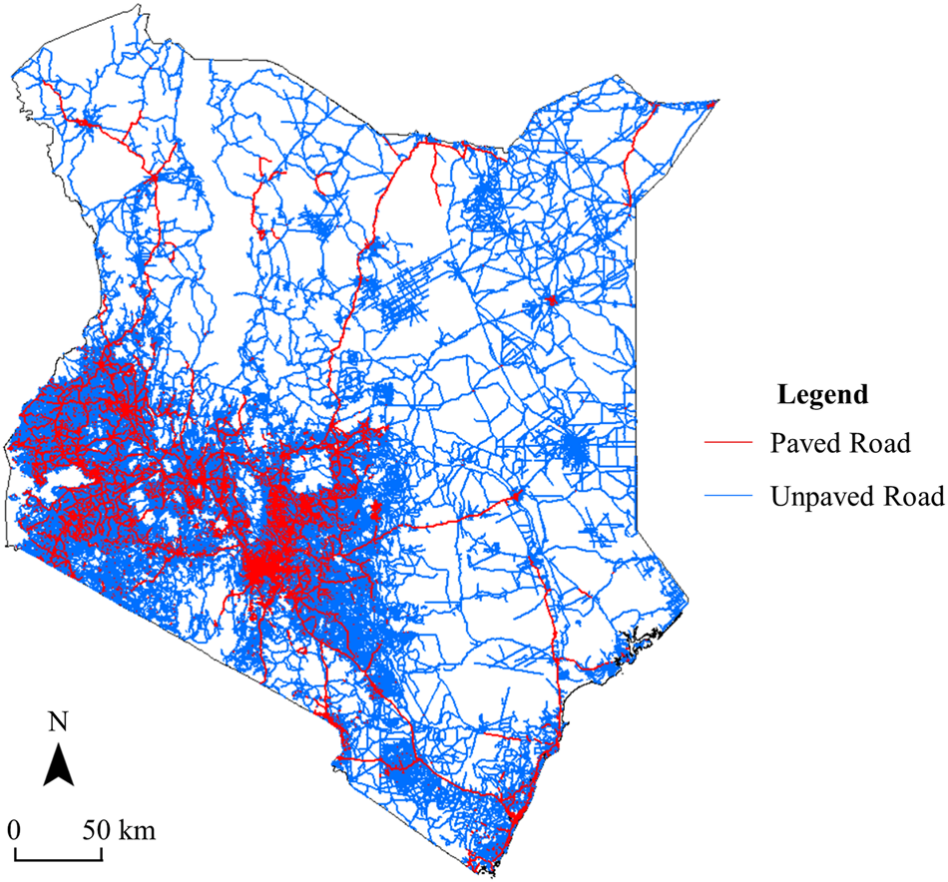
The road surface type map of Kenya.

1. A dataset of road surface type in Kenya was developed in this study (Fig. 4). It was found that most roads in Kenya were unpaved, and the proportion of paved roads in the whole country was only 30%, mainly distributed in the southwestern region of Kenya. There was a significant difference in the road paving rate between urban and rural areas (Fig. 5). Urban areas (Mobass and Nerobi, Fig. 5) had a higher proportion of paved roads, which was much higher than rural areas (Bomet and Busia, Fig. 5), where only a few main roads were paved. The dataset and these findings can provide decision support for the Kenyan government departments to improve road infrastructure.

**Fig. 5:**
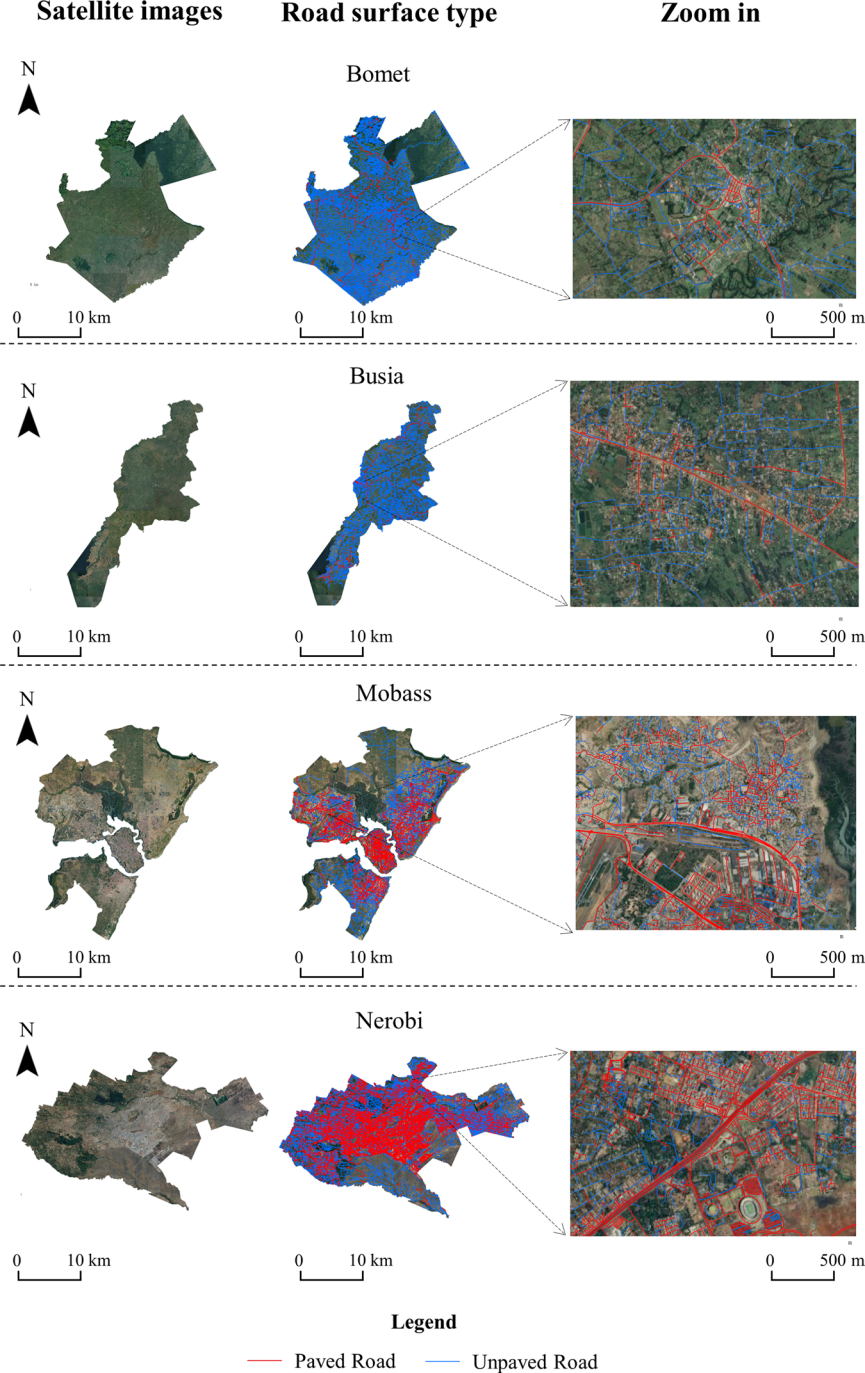
Samples results of the road surface type of Kenya. The first column shows Google satellite images of four counties. The second column shows the mapping result, and the third column zooms to example locations within the city illustrate the road surface type map that we produced.
2. A method for identifying road surface types based on OSM road data and high-resolution Google satellite imagery was proposed in this study. These data can be used for free, so the method may also be applied to other countries and regions to develop data products of road surfaces.
3. Identifying road surface information can help determine all-season roads and assess the SDG 9.1.1 indicator, i.e., rural accessibility. The method of this study can provide data support for assessing this sustainable development indicator, e.g., by combining our dataset with population and urban area datasets^6^. It may also be possible to employ multiple datasets to explore the relationship between road surface type and its impact on road safety, energy consumption, as well as socio-economic development^1–3^.

## Data Availability

The data files and the python scripts used for model training are available online through GitHub repository: https://github.com/Dsayddd/RoadSurface.
